# Genetic diversity of *Salixlapponum* populations in Central Europe

**DOI:** 10.3897/phytokeys.184.71641

**Published:** 2021-11-05

**Authors:** Jacek Urbaniak, Paweł Kwiatkowski, Paweł Pawlikowski

**Affiliations:** 1 Department of Botany and Plant Ecology, Wrocław University of Environmental and Life Sciences, Poland Wrocław University of Environmental and Life Sciences Wroclaw Poland; 2 Institute of Biology, Biotechnology and Environmental Protection, University of Silesia in Katowice, Poland University of Silesia in Katowice Katowice Poland; 3 Department of Plant Ecology and Environmental Conservation, Faculty of Biology, Biological and Chemical Research Centre, University of Warsaw, Poland University of Warsaw Warszawa Poland

**Keywords:** Europe, genetic variation, phylogeography, relict plant, Salicaceae, *Salix*

## Abstract

*Salixlapponum* is a cold-tolerant relict species in Europe that occurs in several sites, probably reflecting previous migration routes of *S.lapponum* during the Pleistocene. However, only a few data are available on the genetic structures of populations of *S.lapponum*. In this study, we use PCR-ISSR markers to investigate genetic variation in 19 European populations of *S.lapponum* L. AMOVA analysis shows that most of the variation (55.8%) occurs within populations; variability among groups accounts for 19.7%. An AMOVA analysis based on four groups determined by STRUCTURE analysis shows similar results: variability of 54.1% within the population and variability of 18.9% between the four population groups, based on geographic regions. Within individual geographic groups, which are characterised by the studied populations, the lowest variability (as well as the highest homogeneity) was found in populations located in Belarus. The obtained results are consistent with our expectations that the European Lowland could be a significant geographic barrier for gene flow over large geographic distances for *S.lapponum*. Both the Scandinavian and Belarusian populations, as well as those coming from NE Poland, are characterised by significant genetic distinctiveness. However, some populations from NE Poland and the Sudetes show similarities with populations from other geographic regions, indicating existing genetic relationships between them. Moreover, the results suggest a fairly clear division of the population into 4 emerging geographic regions, although separated by a geographical barrier: the Polish lowland, which forms part of the larger geographic unit known as the European Lowland.

## Introduction

Quaternary glaciation, with numerous glaciations and deglaciations in Scandinavia and Central Europe, strongly influenced changes in the distributions of both specific plants and entire plant biomes. During Pleistocene climatic fluctuations, numerous arctic plants migrated southward, where they commonly colonised habitats primarily in European mountain ranges; in some cases, they also disappeared from these locations during warmer period called interglacials. Currently, various plant species growing in boreal areas in the Northern Hemisphere and in subalpine zones in lower latitudes in isolated mountains of the alpine system (subalpine zones) or on the lowlands – often grow on the edges of their ranges, presenting disjunct geographic distribution (Comes and Kadereit 2003; Schönswetter et al. 2003, 2005; Urbaniak et al. 2019).

Disjunctive plant populations are often attributed to isolated localities in specific habitats where the climatic and edaphic conditions have allowed for their survival as relict species until the present postglacial period (Birks 2008; Nagamitsu et al. 2014; Urbaniak et al. 2018a). The history of plant relicts relates primarily to the period of Pleistocene glaciations and climatic fluctuations. During the Ice Age, the tundra developed on a portion of the European Lowlands (Van Andel 2002; Birks and Willis 2008), where arctic and alpine plants grew on much wider territory than their current ranges. During each glaciation, species extended their ranges to lowland areas. In warmer interglacial periods, they were crowded out and retreated to specific refugia in higher mountain locations, such as alpine and subalpine zones or specific sites such as peat bogs in lowlands. Plant species that lived outside of refugia retreated northward or disappeared completely. Under these conditions, gene flow and some isolated populations could occur in between migrating populations; these were commonly also subjected to unfavourable genetic conditions such as a sharp reduction in population size due to environmental events, which reduced variation in the gene pool (known as a population bottleneck) or due to other changes in allele frequency, including genetic drift (Comes and Kadereit 1998; Hewitt 2004; Schönswetter et al. 2005; Stewart et al. 2010; Urbaniak et al. 2019). On the other hand, the effect of plant disjunction on genetic diversity and genetic structure can vary in ways that are not fully understood. However, some papers that have examined the effect of spreading disjunctive plant populations have clearly reported its negative effect (Paun et al. 2008).

Numerous phylogeographic studies have described processes including colonisations or re-colonisations from refugial zones, extinctions, or migrations across thousands of kilometres in the Northern Hemisphere (Schönswetter et al. 2004; Oliver et al. 2006). In recent years, intensive studies have sought to explain the origins of disjunct taxa (which originate from the Arctic and from alpine flora and trace their postglacial migratory routes using various molecular tools, including AFLP techniques, microsatellite markers, or chloroplast DNA sequencing (Abbott et al. 2000; Abbott and Comes 2003; Skrede et al. 2006; Alsos et al. 2009; Allen et al. 2015). Previous research has highlighted the lack of a common model for the migration of arctic-alpine plants, even for species with similar habitat requirements (Skrede et al. 2009; Ronikier 2011).

During the Last Glacial Maximum (LGM), all natural vegetation of temperate Europe changed substantially. This also applies to the development history of various *Salix* species, for which results have been obtained from numerous paleobotanical studies and from research using modern genetic methods (Palmé et al. 2003; Reichs et al. 2007; Stamati et al. 2007; Alsos et al. 2009; Sochor et al. 2013; Berlin et al. 2014; Mirski et al. 2017; Wagner et al. 2018; Gouker et al. 2019; Hao et al. 2019; Liu et al. 2020; Wagner et al. 2020). In general, *S.lapponum* L. [Sp. Pl. 1019, 1753] belongs to sect. Villosae (Andersson) Rouy, subgenera *Vetrix* Dumort. (Rechinger and Akeroyd 1993; Argus 1997; Skvortsov 1999). It is a typical Euro-Siberian, boreal species, with a disjunctive arctic-alpine type of geographical distribution (Jalas and Suominen 1976; Hultén and Fries 1986; Stamati et al. 2007). *S.lapponum* can be found primarily in northern Europe and western Siberia and in isolated localities detached from the main distribution range in the mountains of central and southern Europe (the Auvergne, Pyrenees, Sudetes, Carpathians and Rhodopes). For these reasons, *S.lapponum* L. like *Andromedapolifolia* L., *Betulanana* L., *Carexbigelowii* Torr. & Schwein., *Dryasoctopetala* L., *Juncustrifidus* L., *Rubuschamaemorus* L., *Salixherbacea* L. and *Saxifragaoppositifolia* L.– is considered a glacial relict (Dahl 1998; Kwiatkowski and Krahulec 2016; Dítě et al. 2018). This species grows on open or only partially shaded wet places, peat bogs, swamps, meadows at the banks of lakes and streams. It is a component of various types of vegetation such as *Caricionnigrae*, *Rhynchosporionalbae*, *Molinioncaeruleae* or *Adenostyllionalliariae*, *Salicionsilesiacae* (Dierssen 1996; Kołos et al. 2015; Hroneš et al. 2018). It is a diploid species (2n = 38; Chmelař 1979). The expansion of willows has been facilitated by their ability to settle in appropriate niches, which are often small enclaves among other biotopes (Abbott et al. 2000; Birks and Willis 2008). Furthermore, due to the characteristic area of distribution of *S.lapponum* in Europe, its presence throughout diverse altitudes (lowlands, mountain areas, in subalpine zones) and its fairly narrow stenotypic habitats (wet meadows, peat bogs, and springs), *S.lapponum* constitutes a good model species for explaining the complex processes that lead to the formation of arctic-alpine disjunction. Thus, we pursue three aims in this study: 1) to determine the degree of genetic similarity between populations of *S.lapponum*; 2) to explain the origin of Central European populations situated in the contact zone with the main range and in isolated refugia; 3) to describe probable migration routes.

## Material and methods

### Study regions and sampling of the populations

**Figure 1.**
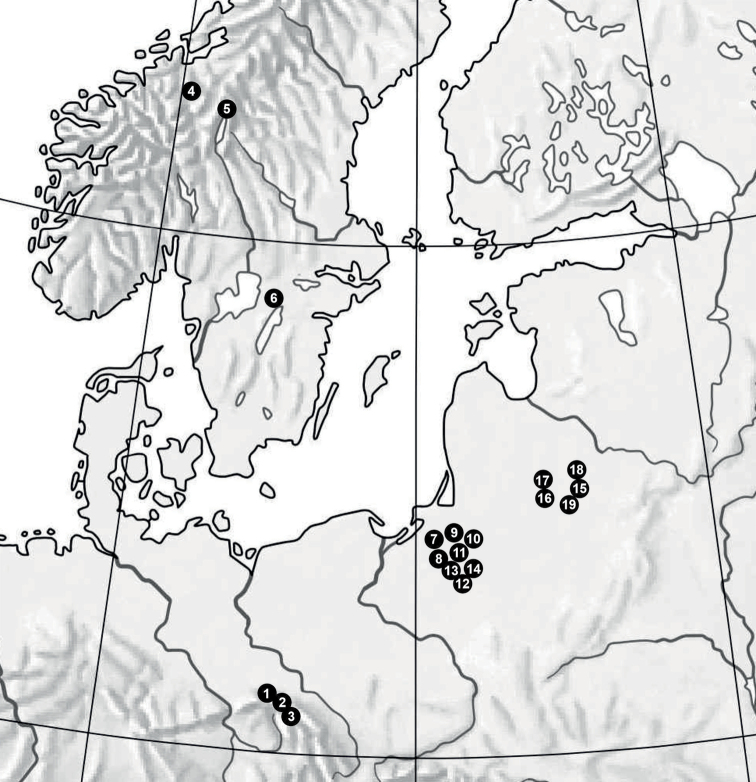
Location of the studied populations of *S.lapponum*. Population abbreviations are the same as in Table 1.

For this study, we used samples collected from nineteen populations of species across the geographical range in Europe (Table 1, Fig. 1).

**Table 1.** Populations of *S.lapponum* included in the study: location in geographical regions, coordinates and Nei’s gene diversity value. The populations’ abbreviations are given below the table.

Region	Population (number) / abbreviation^1^	Longitude, Latitude	h = Nei’s (1973)
Sudetes	(1) KWS	50°45'22"N, 15°41'29"E	0.144
	(2) POL	50°46'5"N, 15°42'35"E	0.136
	(3) KMS	50°44'51"N, 15°42'0"E	0.147
Scandinavia	(4) DOV	62°11'29"N, 9°44'59"E	0.143
	(5) FUL	61°29'58"N, 12°42'34"E	0.144
	(6) NAT	59°21'42"N, 15°8'52"E	0.121
NE Poland	(7) DWO	53°56'31"N, 23°23'16"E	0.172
	(8) WIZ	53°11'48"N, 22°23'45"E	0.110
	(9) SZT	54°7'55"N, 23°24'10"E	0.139
	(10) PRU	54°9'51"N, 22°55'24"E	0.098
	(11) BKL	53°17'9"N, 22°36'22"E	0.114
	(12) BIA	52°41'22"N, 23°44'46"E	0.147
	(13) BLA	53°16'32"N, 22°32'58"E	0.162
	(14) JMO	51°27'36"N, 23°7'14"E	0.109
Belarus	(15) GLU	55°7'38"N, 27°43'5"E	0.129
	(16) DIK	55°32'26"N, 27°50'1"E	0.163
	(17) SIE	55°47'25"N, 29°3'31"E	0.140
	(18) WIT	55°13'21"N, 29°0'11"E	0.122
	(19) BEN	55°33'37"N, 29°14'7"E	0.121

^1^ KWS – Kocioł Wielkiego Stawu (Karkonosze Mts.), POL – Polana (Karkonosze Mts.), KMS – Kocioł Małego Stawu (Karkosze Mts.), DOV – Dovre (Dovre Mts.), FUL – Fuljaflaet (Fuljaflaet Mts.), NAT – Natsverde (Natsverde Mts.), DWO – Dworczyska (Suwałki Region), WIZ – Wizna (Suwałki Region), SZT – Sztabinki (Suwałki Region), PRU – Prudzieniszki (Suwałki Region), BKL – Kładka (Biebrza Region), BIA – Budy (Białystok Region), BLA – Ławki (Biebrza Region), JMO – Lake Moszne (Polesie Region), GLU – Glubokoye (Głębock Region), DIK – Dikoye Blota (Jelnia Region), SIE – Sierewyszcze (Rosson Region), WIT Witebszczyzna (Krasnaje Region), BEN Benczynski Zapowiednik (Połock Region).

Molecular analysis was conducted on plants from all populations. In the field, leaves of *S.lapponum* were randomly sampled in populations at distances that generally depended on the spatial extent of the populations (5–6 m). Fresh leaves were collected in plastic zip bags, dried in silica orange gel, transported to the laboratory, and stored until DNA extraction. In general, we studied eight plant samples per population and 152 specimens of *S.lapponum* in total. Whole plant material was collected in the months of June or July from 2015 to 2017. Herbarium voucher specimens that were used for DNA are stored in author collection (J. U.) deposited at WRSL Herbarium (Wrocław, Poland) and are available on request.

### DNA extraction and molecular analysis

The genomic DNA was isolated after cell disrupting in a Mixer Mill MM400 (Retsch, Haan, Germany), using a DNeasy Plant Mini Kit (Qiagen, Hilden, Germany) according to the manufacturer’s protocol. The quality of the isolated DNA was determined using 1% TBE agarose electrophoresis. The Inter Simple Sequence Repeat microsatellite markers were selected to study the genetic diversity of the populations of *S.lapponum*. These markers are known to be highly polymorphic and are useful in studies on genetic diversity and species relationships; they enable easy differentiation of closely related specimens. ISSR has also been successfully employed to assess hybridisation and to detect hybrid taxa (Ziętkiewicz et al. 1994; Conte et al. 2007; Goldman 2008). These markers have some limitations in use and are less effective than, for example AFLP, but are still used in research. Prior to the study, about 80 ISSR primers were checked for their usefulness in determining population differentiation in *S.lapponum*, based on the experience of Sulima and Przyborowski (2013). A total of twelve primers: ISSR: 2, 3, 4, 5, 91, 92, 93, 94, 95, 137, 139, 142 showed a satisfactory amplification and generated an acceptable number of polymorphic bands (Suppl. material 2). The number of amplified products varied from four to nine within a size range of 100–2.000 bp, depending on the specific primer. PCR reactions were performed in 15-μl reaction tubes that contained a Dream Taq reaction buffer containing MgCl_2_, a 0.2 mM dNTP mix, 1u DreamTaq DNA polymerase (Thermo Fisher Scientific, Waltham, MA, USA), 0.5 mM ISSR primer, and 0.8 μl of total genomic DNA. The PCR cycle consisted of an initial denaturation at 95 °C for 6 min, according to a previous study (Urbaniak et al. 2019), followed by 33 cycles at 95 °C for 30 seconds. The adequate annealing temperature was tested using the gradient method for 30 seconds and a 72 °C elongation for 30 seconds, with a final extension of 10 min at 72 °C. For the PCR reactions, a Veriti Thermal Cycler (Life Technologies, Carlsbad, CA, USA) was used. The PCR ISSR amplification products were separated in 1% agarose gel, photographed, and compared with the DNA mass ruler (Thermo Fisher Scientific Waltham, MA, USA). All laboratory analyses were performed at the Department of Botany and Plant Ecology at Wrocław University of Environmental and Life Sciences.

### Molecular data analysis

The results were analysed using CLIQS software (Totallab 2016). The markers were encoded in a binary matrix and used for computations. The AMOVA (analyses of the molecular variance) were performed using ARLEQUIN 3.5.1 with 1000 permutations to determine the distribution of genetic variation within and among the populations and to assess the importance of the main groups of populations (Excoffier and Lischer 2010). Nei’s genetic identity (Nei 1978) index was calculated using POPGENE v. 1.32 (Yeh et al. 1999). Bayesian clustering was applied using STRUCTURE 2.3.4 (Pritchard et al. 2000; Evanno et al. 2005; Falush et al. 2007) based on an admixture model. The numbers of K from two to seven were tested with ten replications per K using 100,000 burn-in iteration followed by 2,000,000 MCMC iterations. Output data with multiple values of K and hundreds of iterations were analysed using STRUCTURE HARVESTER (Earl and Holdt 2012). CLUMPAK software (Kopelman et al. 2015) was used to produce graphical displays of the STRUCTURE 2.3.4 results and to compute the necessary statistics. To reconstruct the relationships among the analysed populations, we used the Neighbour Net approach as implemented in the SPLITSTREE (Huson and Bryant 2006), using 1000 bootstrap replicates.

All scientific names are given following IPNI (2021). The abbreviated author names for plant names are given as in IPNI (2021) following recommendations of ICN (Turland et al. 2018).

## Results

The research markers selected by us for studying (ISSR) have some limitations in their application due to the lower number of generated markers than for example AFLP, but are still widely used in various types of phylogeographic research. Molecular variance analysis (AMOVA) indicates that most of the genetic variation in two groups of populations: Sudetes vs. NE Poland and Belarus occurs within populations (55.8%), while the variability among the groups accounts for 19.7% (Table 2).

**Table 2.** Results of AMOVA analysis studied populations *S.lapponum*.

Groups/ populations	Partitioning	d.f.	Sum of Squares	Variance components	Percentage of variation	Fst-statistic
All populations	Among populations	19	1245.5	7.3	43.8	0.43
	Within populations	134	1255.2	9.3	56.2	
Sudetes	Among populations	3	172.9	5.9	37.4	0.37
	Within populations	28	279.4	9.9	62.6	
Scandinavia	Among populations	2	151.3	8.3	48.2	0.48
	Within populations	21	188.2	8.9	51.8	
NE Poland	Among populations	7	358.7	5.4	37.2	0.37
	Within populations	54	495.1	9.2	62.8	
Belarus	Among populations	4	118.9	2.8	23.1	0.23
	Within populations	31	292.4	9.4	76.9	
Sudetes and NE Poland vs. Belarus	Among groups	1	158.4	3.4	19.7	0.44
	Among populations	7	291.8	4.2	24.5	
	Within populations	59	571.8	9.7	55.8	
Sudetes vs. Scandinavia vs. NE Poland vs. Belarus	Among groups	3	443.6	2.6	18.9	0.46
	Among populations	16	801.9	5.3	30.6	
	Within populations	134	1255.2	9.4	54.1	

The AMOVA analysis based on four groups (Sudetes vs. Scandinavia vs. NE Poland vs. Belarus) shows a similar result: variability of 54.1% within the population and variability of 18.9% between the four groups of the population based on geographic regions. High genetic variability was also found with the analysis of all studied populations, which were treated as one group. The genetic variability value was 43.8% among populations and 56.2% within populations; the F_st_ value was 0.43 (p < 0.001). Within geographic groups, which are characterised by the studied populations, the lowest variability and also the highest homogeneity were found in populations located in Belarus. The genetic variation among Belarusian populations was low (23.1%). In contrast, the highest inter-population variability was found in Scandinavia (48.2%) and the variability in the Sudetes and NE Poland was similar: 37.4% and 37.2%, respectively.

High and statistically significant genetic differences between all geographical groups of the population were found; again, the greatest differentiation was noted between the Belarusian populations and the Scandinavian populations (0.5). The greatest differences were noted between the populations from Belarus and between the populations from the Sudetes and NE Poland (Table 3); these were also statistically significant. Based on the obtained results, we also calculated the genetic diversity of the studied populations of *S.lapponum* in Europe.

**Table 3.** Pairwise genetic differentiation in between group of populations *S.lapponum* studied using ISSR microsatellites. Number of studied populations in each groups are given in brackets in the first column.

	All Groups	Sudetes	Scandinavia	NE Poland	Belarus
All Groups _(19)_	0.46				
Sudetes _(3)_		-			
Scandinavia _(3)_		0.43	-		
NE Poland _(8)_		0.40	0.44	-	
Belarus _(5)_		0.47	0.50	0.49	-

Both the highest and the lowest index of genetic diversity, based on Nei’s (1978) index, were found in the geographic region of NE Poland, reaching the highest value in the population from Dworczyska (DWO) (0.172) and the lowest value in the population located in Prudzieniszniki (PRU) (0.098). Correlation analysis of the relationship between geographic and genetic distances in the study area did not show any significant correlation between genetic variation and the geographic distance between the studied populations (r = 0.121; *p* = 0.186).

**Figure 2.**
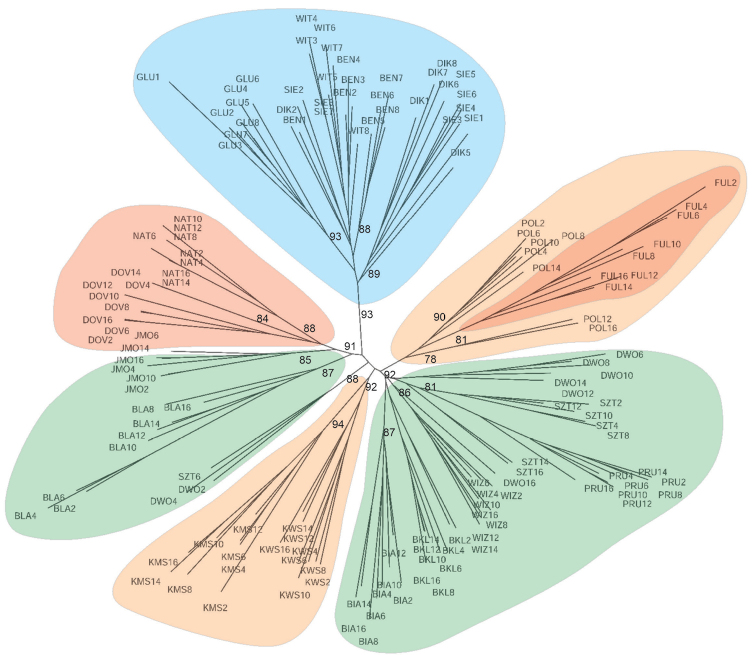
Neighbor-Net of *S.lapponum* individuals based on Nei (1973) coefficient. Population abbreviations are the same as in Table 1.

The analysis using neighbourhood joining for reconstructing the relationships among populations, revealed differences between the population samples collected from different geographical regions of *S.lapponum*’s distribution (Fig. 2). The studied populations were grouped according to their geographic distribution, creating separate groups.

Moreover, all of the selected subgroups were well-supported by bootstrap analysis. Populations from Belarus (GLU, DIK, SIE, WIT, BEN) showing a high similarity with each other (Fig. 2) were included in one of the distinguishing groups on the figure. Plants from populations collected in NE Poland (DWO, WIZ, PCS, PRU, BKL, BIA, BLA, JMO) are divided into two groups, also with quite high bootstrap support, although these groups are partially mixed with each other and some representatives of the population are placed in both groups. A separate group is composed of individuals from the populations located in the Sudetes (KWS, POL, KMS) that are grouped in one cluster on the figure. The exception is a population from Polana (POL), which seems to be genetically similar to the Scandinavian population gathered in Fuljalfleet (FUL). Both populations form a highly distinctive group with high bootstrap support (Fig. 2). The remaining Scandinavian populations form a separate and distinct group.

**Figure 3.**
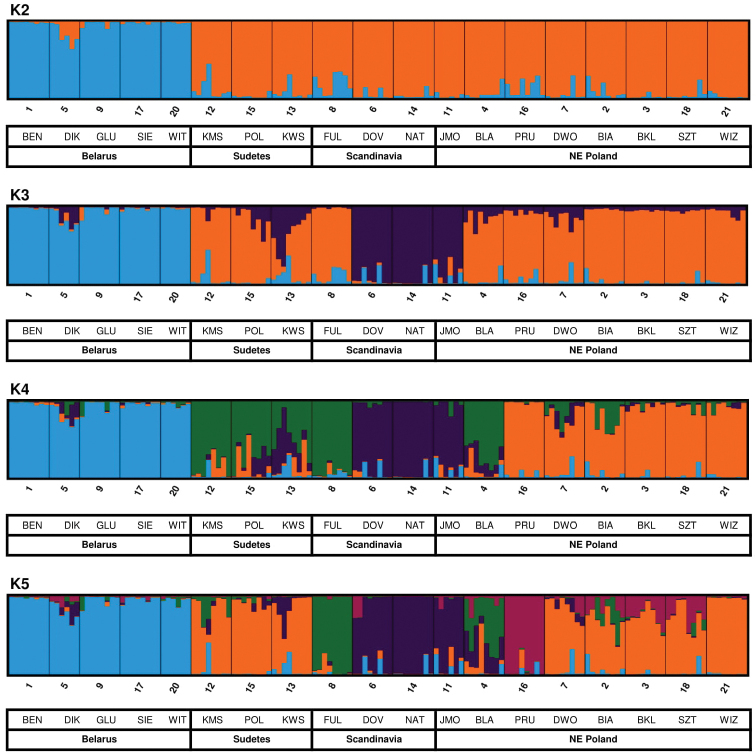
Results of the Bayesian admixture analysis data for populations of *S.lapponum* using STRUCTURE software. Population abbreviations are the same as in Table 1.

Tests performed with the use of the STRUCTURE program, with which the clustering method was implemented, allowed for a more precise elucidation of the genetic variability of the *S.lapponum* population (Fig. 3). The analysis was run for *K* = 2–5; for these results, STRUCTURE HARVESTER showed that the optimal number of populations (*K*) was four, as reported by Evanno et al. (2005), although the highest log-likelihood value was also found for *K* = 5 (Suppl. material 3).

For *K* = 2, all of the populations studied were divided into two groups: populations from Belarus and all other geographical regions (Sudetes, Scandinavia, NE Poland). In general – not only for K = 2 but also for *K* > 2 the populations from Belarus appeared as most distinct in all runs. For *K* = 3, populations from the Sudetes grouped with populations from NE Poland, while Scandinavian populations formed a separate group, except for one population (FUL) that seems to be genetically closer to populations from the Sudetes. For *K* = 5, populations from Belarus and the Sudetes form nearly a consistent group; however, specimens from the Sudetes populations showed closer relations with several populations from NE Poland. Results obtained with the STRUCTURE program are almost identical to the results presented in Fig. 2.

## Discussion

Climate fluctuations during the Pleistocene markedly changed distribution patterns for both plant and animal species. Consequently, full phylogenetic compatibility of species can be found only rarely, and usually only on a regional scale (Freeland 2008). When genetic research covers a much larger area, the phytogeographic variability becomes much more complex and is usually closely correlated geographically with the processes of Pleistocene glaciation (Taberlet et al. 1998; Hewitt 2004; Lindner et al. 2004; Parmesan 2006). This is the case with many species, including glacial relics occurring in isolated sites such as mountains or in the Polish lowlands (Urbaniak et al. 2018b). The ability of *S.lapponum* to survive in such unfavourable conditions in isolated regions, such as glacial refuges, was very important for its later recolonisation and the spread of genetic lines during the interglacial period. However, the reconstruction of the glacial and postglacial history of *S.lapponum* on the basis of palaeoecological data is quite difficult, because the record of macrofossils is not known (Ralska-Jasiewiczowa et al. 2004). Fragmentation of the species occurrence area substantially reduces genetic diversity and increases inter-population genetic divergence, mostly by limiting the flow of alleles among fragmented populations, which thereby increases inbreeding and the likelihood of genetic drift in the population. Consequently, this can lead to the extinction of the species due to the reduction of allele diversity in the population, which decreases the adaptability of the species.

The results obtained from genetic analyses of the *S.lapponum* population show that genetic diversity between the studied populations in the area of the species occurrence is significant, while gene flow is clearly geographically limited. This fact may be explained by the lack of continuity in the occurrence of *S.lapponum* and by the large geographic areas that separate the studied populations, thus preventing the free flow of genes. For example, in central Poland, there are no *S.lapponum* sites – not even scattered sites. As can be seen on Fig. 2, populations from Belarus form separate clusters. It appears that this group of populations seems to be distinct, showing high genetic distinctiveness from populations from other geographic regions, while also showing low inter - population variability (Fst = 23.1, Suppl. material 1). The STRUCTURE analyses (K = 2) show that populations from Belarus are most separated or isolated from the other populations. Analyses at higher K indicate some similarities between the remaining populations, expressed particularly in the form of allele transmission between them (Fig. 3). These research results also indicate a fairly high gene admission between the populations from NE Poland and other geographical regions, including Scandinavia, although some differences in genetic variation between them are also apparent. Similar results confirming the isolation of distant populations of *S.alba* are given by Puyvelde and Triest (2007) from the Alp region, and by Sulima and Przyborowski (2013) regarding *S.purpurea* from NE Poland.

The populations of *S.lapponum* in Scandinavia seem to be different from those in the rest of Europe, apart from the Fuljafleat (FUL) population. At the same time, the effects of between populations in Scandinavia and other regions are visible. In Scandinavian populations, however, there are alleles common to populations from both the Sudetes and NE Poland, demonstrating close genetic relationships and similarities between them (Fig. 2, 3). The population of Fuljafleat (FUL) is definitely most closely related to the populations of the Sudetes (K = 3–4) and NE Poland (K = 3–5). It is possible that the migrating individuals of *S.lapponum* remained in relatively small populations and, therefore, the combined effects of genetic drift and intra-population mutational processes increased differentiation between them. This is indicated by significant genetic diversity, as illustrated by the very high calculated coefficients of differentiation between the studied populations of *S.lapponum* presented in Tables 2, 3, Suppl. material 1.

There are also similarities between the analysed populations from the Sudetes and NE Poland, which may be attributed to the relatively small populations compared; this is confirmed by the results of the pairwise genetic differentiation analysis (Table 2). The calculated value between the populations from the Sudetes and NE Poland was the lowest (0.40) among all analysed population groups. This may result from a common history and species migration, especially regarding the migration of *S.lapponum* from Scandinavia to the south, which occurred during the favourable climatic periods of the Pleistocene through Poland, which was almost certainly favoured by the climatic conditions at the time. In the Sudetes, *S.lapponum* reaches its southern distribution limit, and the obtained results indicate closer relations between populations from the Sudetes and those from NE Poland. Certainly, in the past, isolated refugia existed in Central Europe. It is also possible that the separation of both population groups from the Sudetes and NE Poland took place relatively recently. Similar results have also been obtained for *S.arbutifolia* from the most NW locations in Japan. Nagamitsu et al. (2014) report a large genetic divergence between the distant geographic barrier populations between Sakhalin and Hokkaido, which entails a complicated history of migration and colonisation of this species. The most extreme populations had the lowest genetic diversity and were the most distinct from the rest. Therefore, the low genetic diversity and high genetic diversity on the extreme of ranges may suggest a significant influence of genetic drift on the genetic structure of separated populations – not only of *S.lapponum*, but also of other species.

In the Northern Hemisphere, climatic oscillations during the Quaternary period caused significant changes in plant distribution, which resulted in the repeated expansion and fragmentation of species’ ranges and affected their patterns of genetic diversity. Cold-adapted plants (arctic and boreal) are believed to be more threatened during the Quaternary period than other plant groups (Comes and Kadereit 1998; Alsos et al. 2012; Eidesen et al. 2013; Paulus et al. 2013).

The currently observed decline in population sizes and geographical ranges, limited generative reproduction, and short-range spreading make boreal species more susceptible to loss of genetic diversity than, for example, plants in the temperate or Mediterranean zones. Moreover, climate scenarios (Crawford 2008) predict that the geographic range of northern species will shrink and move northwards or to higher altitudes, leading to greater isolation of their populations, or even extinction. The currently preserved disjunctive geographic range of this glacial relic is certainly related to Weichselian glaciation. Originating from the northern part of Europe and Asia, *S.lapponum* probably survived the glaciation in several isolated locations in Central Europe. Therefore, for example, populations from the Sudetes can be included in the populations found in the interglacial refugia for cold-adapted species (Lister et al. 2010; Tzedakis et al. 2013). During successive glaciations, *S.lapponum* probably colonised the areas of Belarus and NE Poland, far away from the population in the Sudetes and relatively close to the Scandinavian sites.

The clearly low and similar level of variation, especially within Belarusian populations, may result from two different processes: the founding effect and genetic drift caused by small population sizes. However, in both the Scandinavian and Sudeten populations, probably there was increased genetic variation, represented in migrants who re-settled the newly available space. In many plant species, a correlation is found between population size and genetic variation (Gaudeul et al. 2000; Despres et al. 2002), which is consistent with the hypothesis that small populations cannot maintain high genetic variation like larger populations. The high genetic diversity can be also explained by the presence of glacial refuges in the past or by the presence of a specific contact zone with different phylogenetic lines. One of these zones is Central and Eastern Europe (Taberlet et al. 1998; Hewitt 1999). Therefore, it is likely that *S.lapponum* could survive the LGM in NE Poland and Belarus on the edge of the Scandinavian glacier in the area with various phylogenetic lines. It is also possible that this part of Europe was re-occupied by the migration waves of *S.lapponum* originating in Scandinavia and the Sudetes.

These two waves of migration probably met, creating a local suture zone with a mixed haplotype character. A similar scenario has also been proposed for *Populustremula* L. in Central Europe (Fussi et al. 2010) and for the numerous populations of *Salixherbacea* (Alsos et al. 2009). However, to reach a more thorough understanding, additional studies are needed that include not only intra- and inter-population variability studies but also cpDNA-based phylogenetic lineage studies. Results obtained with this method could further elucidate similarities or differences in the DNA haplotypes of *S.lapponum*.

## Conclusions

The conducted research shows the division of the studied *Salixlapponum* populations into several genetic groups. The populations from Belarus were the most genetically different. Populations from NE Poland and the Sudetes show similarities with populations from other geographic regions, indicating existing genetic relationships between them. It is possible that there was a meeting and exchange of genes between populations in southern Europe and Scandinavian populations and from NE Poland. However, to reach a more thorough understanding, additional studies are needed that include not only intra- and inter-population variability studies based on investigating phylogenetic lineage.

## Supplementary materials

Supplementary material 1 Table S1 Data type Docx file. Explanation note Parwise Nei’s genetic distances (Nei, 1978) for populations of *S.lapponum* based on ISSR analysis. https://binary.pensoft.net/file/606706This dataset is made available under the Open Database License (http://opendatacommons.org/licenses/odbl/1.0/). The Open Database License (ODbL) is a license agreement intended to allow users to freely share, modify, and use this Dataset while maintaining this same freedom for others, provided that the original source and author(s) are credited.Jacek Urbaniak, Paweł Kwiatkowski, Paweł Pawlikowski

Supplementary material 2 Table S2 Data type Docx file. Explanation note Nucleotide sequences and annealing temperatures of ISSR primers used in study. https://binary.pensoft.net/file/606707This dataset is made available under the Open Database License (http://opendatacommons.org/licenses/odbl/1.0/). The Open Database License (ODbL) is a license agreement intended to allow users to freely share, modify, and use this Dataset while maintaining this same freedom for others, provided that the original source and author(s) are credited.Jacek Urbaniak, Paweł Kwiatkowski, Paweł Pawlikowski

Supplementary material 3 Figure S1 Data type Jpg file. Explanation note Delta K values for Structure analysis. https://binary.pensoft.net/file/606708This dataset is made available under the Open Database License (http://opendatacommons.org/licenses/odbl/1.0/). The Open Database License (ODbL) is a license agreement intended to allow users to freely share, modify, and use this Dataset while maintaining this same freedom for others, provided that the original source and author(s) are credited.Jacek Urbaniak, Paweł Kwiatkowski, Paweł Pawlikowski
