# Supplementary material for: Genetic diversity of Salixlapponum populations in Central Europe
Source: PhytoKeys. 2021 Nov 5;184:83–101. doi: 10.3897/phytokeys.184.71641 (PMC8589822; doi:10.3897/phytokeys.184.71641)
Supplement: Supplementary material 1 — Table S1 [file phytokeys-184-083-s001.docx]

Table. S1 Parwise Nei's genetic distances (Nei, 1978) for populations of S. lapponum based on ISSR analysis.

|  | KWS | POL | KMS | DOV | FUL | NAT | DWO | WIZ | SZT | PRU | BKL | BIA | BLA | JMO | GLU | DIK | SIE | WIT | BEN |
| --- | --- | --- | --- | --- | --- | --- | --- | --- | --- | --- | --- | --- | --- | --- | --- | --- | --- | --- | --- |
| KWS  KWS | - |  |  |  |  |  |  |  |  |  |  |  |  |  |  |  |  |  |  |
| POL | 0.078 | - |  |  |  |  |  |  |  |  |  |  |  |  |  |  |  |  |  |
| KMS | 0.120 | 0.155 | - |  |  |  |  |  |  |  |  |  |  |  |  |  |  |  |  |
| DOV | 0.104 | 0.151 | 0.203 | - |  |  |  |  |  |  |  |  |  |  |  |  |  |  |  |
| FUL | 0.199 | 0.165 | 0.193 | 0.202 | - |  |  |  |  |  |  |  |  |  |  |  |  |  |  |
| NAT | 0.164 | 0.190 | 0.219 | 0.121 | 0.277 | - |  |  |  |  |  |  |  |  |  |  |  |  |  |
| DWO | 0.113 | 0.098 | 0.146 | 0.150 | 0.182 | 0.131 | - |  |  |  |  |  |  |  |  |  |  |  |  |
| WIZ | 0.106 | 0.114 | 0.159 | 0.171 | 0.218 | 0.182 | 0.080 | - |  |  |  |  |  |  |  |  |  |  |  |
| SZT | 0.117 | 0.133 | 0.156 | 0.157 | 0.229 | 0.156 | 0.082 | 0.085 | - |  |  |  |  |  |  |  |  |  |  |
| PRU | 0.221 | 0.243 | 0.251 | 0.204 | 0.247 | 0.245 | 0.159 | 0.176 | 0.126 | - |  |  |  |  |  |  |  |  |  |
| BKL | 0.140 | 0.128 | 0.190 | 0.166 | 0.185 | 0.197 | 0.096 | 0.117 | 0.098 | 0.158 | - |  |  |  |  |  |  |  |  |
| BIA | 0.154 | 0.166 | 0.139 | 0.212 | 0.159 | 0.212 | 0.079 | 0.099 | 0.110 | 0.156 | 0.106 | - |  |  |  |  |  |  |  |
| BLA | 0.180 | 0.140 | 0.154 | 0.161 | 0.190 | 0.177 | 0.146 | 0.182 | 0.157 | 0.200 | 0.151 | 0.153 | - |  |  |  |  |  |  |
| JMO | 0.138 | 0.145 | 0.199 | 0.108 | 0.264 | 0.138 | 0.147 | 0.141 | 0.161 | 0.182 | 0.160 | 0.170 | 0.141 | - |  |  |  |  |  |
| GLU | 0.228 | 0.234 | 0.242 | 0.276 | 0.255 | 0.278 | 0.196 | 0.203 | 0.218 | 0.256 | 0.228 | 0.200 | 0.259 | 0.249 | - |  |  |  |  |
| DIK | 0.179 | 0.212 | 0.193 | 0.198 | 0.234 | 0.208 | 0.178 | 0.187 | 0.140 | 0.195 | 0.185 | 0.176 | 0.185 | 0.189 | 0.090 | - |  |  |  |
| SIE | 0.172 | 0.230 | 0.189 | 0.230 | 0.236 | 0.234 | 0.181 | 0.200 | 0.169 | 0.218 | 0.240 | 0.183 | 0.231 | 0.229 | 0.109 | 0.075 | - |  |  |
| WIT | 0.216 | 0.238 | 0.234 | 0.253 | 0.202 | 0.265 | 0.192 | 0.236 | 0.215 | 0.246 | 0.262 | 0.227 | 0.262 | 0.284 | 0.101 | 0.101 | 0.089 | - |  |
| BEN | 0.176 | 0.219 | 0.210 | 0.217 | 0.222 | 0.218 | 0.168 | 0.181 | 0.186 | 0.230 | 0.209 | 0.193 | 0.262 | 0.223 | 0.063 | 0.071 | 0.068 | 0.065 | - |
