# Supplementary material for: Genetic diversity of Salixlapponum populations in Central Europe
Source: PhytoKeys. 2021 Nov 5;184:83–101. doi: 10.3897/phytokeys.184.71641 (PMC8589822; doi:10.3897/phytokeys.184.71641)
Supplement: Supplementary material 2 — Table S2 [file phytokeys-184-083-s002.docx]

Table S2. Nucleotide sequences and annealing temperatures of ISSR primers used in study

| Primer | Nucleotide sequence | Annealing temperature |
| --- | --- | --- |
| ISSR 2  ISSR 3  ISSR 4  ISSR 5  ISSR 91  ISSR 92  ISSR 93  ISSR 94  ISSR 95  ISSR 137  ISSR 139  ISSR 142 | GGT ACAACAACAACAACA GGCACAACAACAACAACA GAGACAACAACAACAACA GATACAACAACAACAACA  GAGGTGTGTGTGTGTGTO GAAGTGTGTGTGTGTGTG GACGTOTGTGTOTGTGTG AAGGTGTOTGTGTGTGTG AAAGTGTGTOTGTGTGTG GCAACACACACACACAC ACGACACACACACACAC TCGACACACACACACAC | 49  49  49  49  58  59  58  54  65  65  63  64 |
