# Supplementary figures and images for: Genetic diversity of Salixlapponum populations in Central Europe
Source: PhytoKeys. 2021 Nov 5;184:83–101. doi: 10.3897/phytokeys.184.71641 (PMC8589822; doi:10.3897/phytokeys.184.71641)

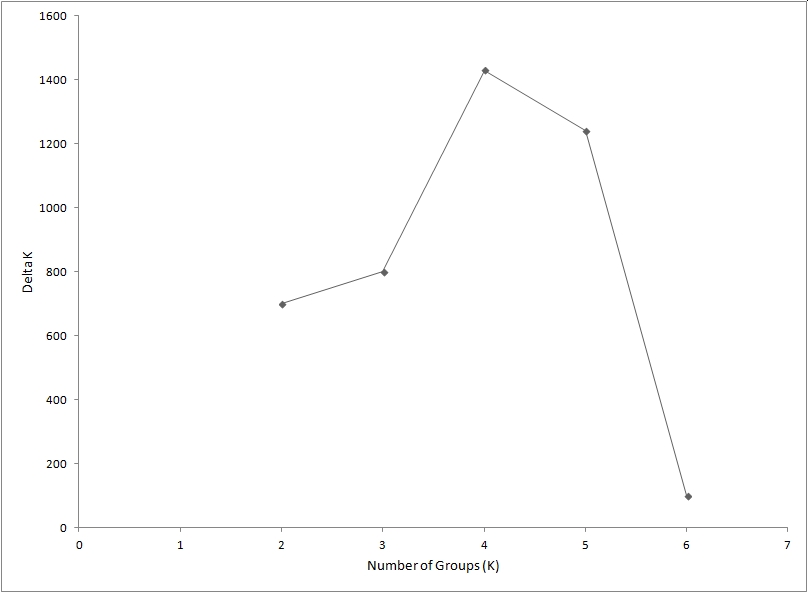

Supplement: Supplementary material 3 — Figure S1 [file phytokeys-184-083-s003.jpg]
